# An Open Pharma Vision for company-sponsored biomedical research publications

**DOI:** 10.1136/bmjopen-2025-104128

**Published:** 2026-05-08

**Authors:** Joana Osório, Slávka Baróniková, Sally-Anne Dews, Santosh Mysore, Kalliopi Patrikis, Valérie Philippon, Christopher P Rains, Anja Schmidt, Catherine Skobe, Christopher C Winchester

**Affiliations:** 1 Oxford PharmaGenesis, Oxford, UK; 2 Alfasigma Belgium BV, Mechelen, Belgium; 3 Pfizer Ltd, Tadworth, UK; 4 GSK, Wavre, Belgium; 5 Gilead Sciences Inc, Foster City, California, USA; 6 UCB, Cambridge, Massachusetts, USA; 7 CPR BioPharma Consulting, Newburyport, Massachusetts, USA; 8 Takeda Development Center Americas Inc, Cambridge, Massachusetts, USA; 9 Pfizer, New York City, New York, USA

**Keywords:** Patient Participation, Clinical Trial, ETHICS (see Medical Ethics)

## Abstract

Open Science aims to fight misinformation and improve trust in scientific research; it encourages the reliability and accessibility of evidence, reduces inequalities through the democratisation of scientific knowledge and focuses scientific endeavours on issues of societal significance. As a multisponsor collaboration committed to driving positive change, Open Pharma has a multifaceted vision for scientific research publications funded by pharmaceutical companies (‘company research publications’) that aligns strongly with Open Science tenets. This new vision statement outlines our forward-looking principles for company research publications, both for short-term attainment (‘immediate’) and long-term commitment (‘ultimate’). Together, the principles provide a framework for positive collective action by all stakeholders involved in the development and dissemination of peer-reviewed company research publications. Underpinned by our central commitment to transparency for company research publications, we outline goals for: universal access to these publications; provision of peer-reviewed plain language summaries of the publications to aid comprehension among non-specialist readers; leveraging author and institutional metadata to advance transparency, discoverability and research impact; working towards FAIR (Findability, Accessibility, Interoperability, and Reuse) data principles through cross-sector consensus and action, and disclosure of patient involvement in research and its reporting to support transparency and encourage a research ecosystem attuned to patient centricity. We call on all stakeholders to realise the Open Pharma Vision and achieve an open and trusted future for company research publications that will ultimately advance patient care and improve global health.

**STRENGTHS AND LIMITATIONS OF THIS STUDY**

- The stakeholders consulted during the 2023 Open Pharma Summit on their aspirations for company research publications represented diverse groups, including:
  - pharmaceutical company professionals working in publications and patient engagement,
  - representatives from publishers and publisher societies,
  - representatives from philanthropic, Open Science, patient advocacy and charity organisations.
- The taskforce who created the vision statement and who are authors of this article comprised experts in biomedical research communications with extensive experience in scientific and medical publications, medical affairs, patient affairs and patient advocacy gained through time working in pharmaceutical companies and in scholarly publishing, and as authors.
- The vision is underpinned by core Open Science tenets, but considers these (for the first time) in the specific context of company-sponsored biomedical research publications.
- The vision statements were developed to include a temporal component to support short-term goal attainment and guide longer-term commitments; practical steps are included to help different stakeholder groups implement positive progress (separately and collectively).
- This article does not specifically address potential barriers to implementing the vision or strategies to overcome them; rather, it is intended to set the stage for further discussion.
- Beyond their contribution at the initiation of the vision development, patients and members of the public were not directly involved in shaping the statements presented in this article.
- No formal consensus method was used by the taskforce to develop the statements.
- As with all consensus outputs, the vision reflects the opinions and experiences of the authors.

## Introduction

 Open Pharma is a multisponsor collaboration committed to driving positive change in the publication of research funded by pharmaceutical companies (‘company research publications’).^1^ We believe that peer-reviewed medical journal publications provide the definitive and trusted record of why and how health research was conducted, what it discovered and what it means. For the stakeholders involved in healthcare decision-making, there is a need for high-quality, peer-reviewed evidence to be findable, comprehensible and actionable by anyone who needs it, anywhere in the world.

Our principles align strongly with those of the Open Science movement and its aspiration to make science more transparent, inclusive and democratic.^2^ Open Science aims to improve the reliability and accessibility of evidence, and trust in scientific research.^2^ It also encourages new social actors to engage in the scientific process to help broaden knowledge, address systemic inequalities, fight misinformation and focus on problems of global societal importance.^2^

**Figure 1:**
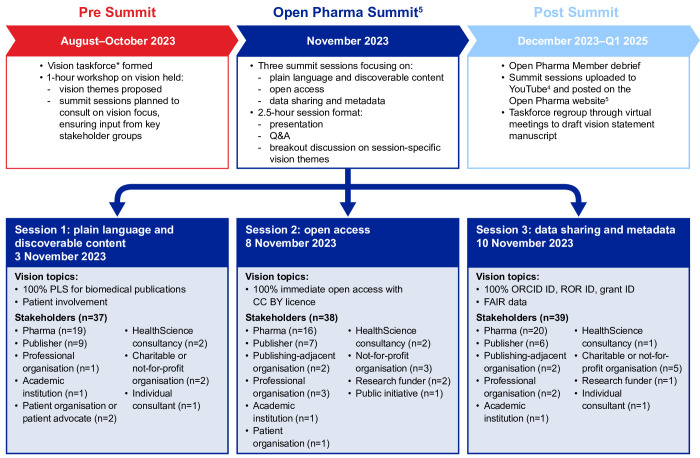
Timeline and stakeholder consultation process followed for the development of the Open Pharma Vision. *The taskforce comprised senior professionals from the pharmaceutical and medical communications sectors who had extensive experience in scientific and medical publications, medical affairs, patient affairs and patient advocacy. CC BY, Creative Commons Attribution; FAIR, Findability, Accessibility, Interoperability, and Reuse; ORCID, Open Researcher and Contributor; PLS, plain language summary/ies; ROR, Research Organization Registry.

We established a taskforce to define steps to advance transparency in company research publications beyond public disclosure of human trial data, which most pharmaceutical companies have already committed to.^3^ The taskforce comprised senior professionals from the pharmaceutical and medical communications sectors who had extensive experience in scientific and medical publications, medical affairs, patient affairs and patient advocacy. Membership of the taskforce was voluntary, and all members were involved in the Open Pharma collaboration. Following consultation with diverse stakeholders during the 2023 Open Pharma Summit on their aspirations for company research publications (figure 1),^4 5^ the role of the taskforce was to formalise and champion the ambitions they had voiced. The Open Pharma Vision distils the principles and actions agreed by the taskforce through virtual and face-to-face meetings and iterative statement refinement, and is informed by the individual opinions and experiences of the authors.

Just as the scope of Open Science extends beyond open access to include transparency, verification of scientific knowledge and collaboration and inclusion,^2^ our vision for company research publications is likewise multifaceted. Here, we outline forward-looking principles for company research publications for short-term attainment (‘immediate’) and long-term commitment (‘ultimate’) (table 1).

**Table 1:** Open Pharma Vision for company research publications

Vision principles	Immediate goal(s)	Ultimate goal
Open access publishing	100% of articles published with any form of OA, including green OA*	100% of papers published with immediate OA under a gold standard CC BY licence
Plain language summaries	100% PLS for all clinical trial publications that underpin regulatory decision-making; minimum PLS standard applied in line with Open Pharma recommendations and published alongside the article abstract† or within supplementary materials	100% PLS for all biomedical research papers and published alongside the article abstract* and in an open access PLS repository
Use of author and institutional metadata	For company authors: mandated use of ORCID iDs and ROR iDs for affiliations For all other authors, contributors and funders: promotion of ORCID iD, ROR iDs for affiliations and grant persistent identifiers (when they exist) to support a year-on-year uptake in use	100% ORCID iDs for all authors; 100% of grant persistent identifiers included in investigator-initiated study publications; 100% ROR iDs for affiliations of all authors and funders
FAIRification of data	Agreement across the pharmaceutical industry about applying the FAIR framework for all data associated with human research publications	All publication-associated data are FAIR while protecting individual privacy
Involving the patient	100% of human research papers include a statement about if/how the patient perspective was included	100% of biomedical research papers include a statement about if/how the patient perspective was integrated in the study and publication concept or plan

* Self-archiving the AAM of VoR, as permitted, in online, public repositories.

† In the full-text PDF of the publication, in the article HTML on the journal website and (for indexed journals) on PubMed.

AAM, author-approved manuscript; CC BY, Creative Commons Attribution; FAIR, Findability, Accessibility, Interoperability, and Reuse; HTML, hypertext markup language; iD, identifier; OA, open access; ORCID, Open Researcher and Contributor; PDF, portable document format; PLS, plain language summary/ies; ROR, Research Organization Registry; VoR, version of record.

Together, the vision statements provide a framework for positive collective action. Our hope is that the Open Pharma Vision not only helps to prioritise and inform our own activities but also serves as a guide for those in the scientific community who are committed to high-quality, high-impact, efficient and reproducible science. Pragmatic implementation steps are also suggested for other key stakeholder groups (tables 2–6).

**Table 2:** Open Pharma Vision and roadmap for open access publication of company research

Vision for open access publishing	
Immediate goal	100% of articles published with any form of OA, including green OA*
Ultimate goal	100% of papers published with immediate OA under a gold standard CC BY licence

Stakeholder actions	
Authors of company-sponsored biomedical research	Endorse the Open Pharma Open Access Position Statement^8^ Request OA and the CC BY option from journals at submission Check whether any (co-)authors can choose an open access option under an institutional agreement when submitting to a peer-reviewed journal Pursue green OA* options when OA is not immediately available through the journal website (eg, share the VoR or AAM via a non-commercial† or commercial‡ public repository, as permitted, but always linking to the journal publication using the DOI URL when citing the work)
Pharmaceutical company funders	Endorse the Open Pharma Open Access Position Statement^8^ Have a publicly accessible OA policy, ideally a mandate registered at the JISC open policy finder database (https://openpolicyfinder.jisc.ac.uk/)§ Request OA and the CC BY option from journals at submission Systematically check whether any authors can choose an open access option in target journals under institutional agreements Consider new business models based on collective or individual company agreements with publishers Share the VoR or AAM via a non-commercial or commercial public repository‡ (green OA),* as permitted, when OA is not immediately available through the journal website, and encourage authors to share the VoR (if available) or the AAM via a non-commercial public repository,† but always linking to the journal publication using the DOI URL when citing the work Explore supporting (and/or signing up to) non-commercial green OA repositories Set and monitor OA targets and reward achievement
Publishers	Endorse the Open Pharma Open Access Position Statement^8^ Allow pharmaceutical companies to publish under the same copyright licences as non-commercial sponsors, including the option to publish CC BY Be transparent about APCs and permissions, including clarity on terms of reuse (eg, what constitutes ‘commercial use’) and the circumstances in which permission will be granted Simplify payment of APCs Consider new business models based on agreements with individual companies or group of companies; these could include read-and-publish deals, subscription-fee replacement fees (eg, transformative agreements); and bundles that may include APC, figure permission and green OA elements Check whether any authors qualify for OA (waived open APCs) under an institutional agreement

* Self-archiving the AAM of VoR, as permitted, in online, public repositories.

† Examples of non-commercial repositories include websites of academic institutions and PubMed Central (https://pmc.ncbi.nlm.nih.gov) or Europe PMC (https://europepmc.org), subject to institutional relationship with PMC/Europe PMC.

‡ Examples of commercial repositories include websites of commercial entities such as pharmaceutical companies.

§ Formerly Sherpa services.

AAM, author-approved manuscript; APC, article processing charge; CC BY, Creative Commons Attribution; DOI, digital object identifier; JISC, Joint Information Systems Committee; OA, open access; PMC, PubMed Central; URL, uniform resource locator; VoR, version of record.

**Table 3:** Open Pharma Vision and roadmap for plain language summaries of company research publications

Vision for plain language summaries	
Immediate goal	100% PLS for all clinical trial publications that underpin regulatory decision-making; minimum PLS standard applied in line with Open Pharma recommendations and published alongside the article abstract* or within supplementary materials
Ultimate goal	100% PLS for all biomedical research papers and published alongside the article abstract* and in an open access PLS repository

Stakeholder actions	
Authors of company-sponsored biomedical research	Adopt the Open Pharma recommendations for PLS for (co-)authored manuscripts^24^ Request budget from grant funders for PLS development by professional writers† Request (eg, in submission covering letters) that journals/publishers peer review PLS, and include them alongside the standard article abstract on PubMed and/or within the publication HTML Make use of PLS and PLSP repositories (eg, organisational/institutional websites and/or third-party content repositories such as Figshare (https://figshare.com) and Kudos (https://www.growkudos.com), pending establishment of additional centralised PLS and/or PLSP platforms)
Pharmaceutical company funders	Adopt the Open Pharma recommendations for PLS for company research publications^24^ Have a public PLS policy, preferably a mandate Implement standard operating procedures and allocate resources to developing PLS Encourage authors and ensure medical writers request that journals/publishers peer review PLS and include them alongside the standard article abstract on PubMed and/or within the publication HTML Make use of PLS and PLSP repositories (eg, organisational/institutional websites and/or third-party content repositories, pending establishment of additional centralised platforms)—linking through to the journal publication using the DOI URL Set and monitor PLS targets and reward achievement
Publishers	Enable the Open Pharma recommendations for inclusion of a (minimum standard) for PLS within the body of published articles and alongside the standard abstract on PubMed Implement standard PLS publication capabilities at their journals Consider dedicating in-house resource to writing PLS Make sure PLS are tagged with the correct metadata for PubMed indexing Measure and report on the impact of publication enhancements, such as graphical abstracts and infographics and multimedia materials that complement the peer-reviewed manuscript (eg, author videos and podcasts) Explore options for provision of PLS in different languages Explore options for development of an open access PLS repository Ensure PLS within the journal publications and standalone PLSP are peer reviewed, and that some of the peer reviewers are non-specialists from target audiences

* In the full-text PDF of the publication, in the article HTML on the journal website and (for indexed journals) on PubMed.

† AI has the potential to provide efficiencies with PLS development; however, it is imperative that the AI-generated PLS be reviewed by a professional medical writer or a representative of the intended non-specialist audience to ensure accuracy and suitability. It is also noteworthy that not all organisations and institutions have access to generative AI capable of PLS development.

AI, artificial intelligence; DOI, digital object identifier; HTML, Hypertext markup language; PDF, portable document format; PLS, plain language summary/ies; PLSP, plain language summary publication; URL, uniform resource locator.

**Table 4:** Open Pharma Vision and roadmap for author and institutional metadata tagging in company research publications

Vision for use of author and institutional metadata	
Immediate goals	For company authors: mandated use of ORCID iDs and ROR iDs for affiliations For all other authors, contributors and funders: promotion of ORCID iD, ROR iDs for affiliations and grant persistent identifiers (when they exist) to support a year-on-year uptake in use
Ultimate goal	100% ORCID iDs for all authors; 100% of grant persistent identifiers included in investigator-initiated study publications; 100% ROR iDs for affiliations of all authors and funders

Stakeholder actions	
Authors of company-sponsored biomedical research	Sign up to ORCID, keep ORCID records up-to-date and include ORCID in all articles; encourage co-authors to do the same Include grant persistent identifiers in publications, whenever possible (ie, where a grant persistent identifier associated with the research is available) Include ROR IDs for affiliated institutes/organisations in all research publications
Pharmaceutical company funders	Mandate, within their publication policy, author agreements and study agreements (including for investigator-initiated studies), the use of ORCID iDs for all authors; highlight this requirement at the time agreements are first shared Ensure persistent identifiers are assigned to all grants provided for investigator-initiated studies and mandate their use in research publications within the investigator agreements Strongly encourage inclusion of ROR IDs (where available) for affiliations of all contributors and funders in all articles
Publishers	Work to improve the ease with which authors can include ORCID iDs as metadata (eg, without having to link/sign in to the ORCID website through journal submission systems) Make ORCID iDs mandatory for all authors Enable grant PIDs and ROR IDs for pharmaceutical companies at their journals

iD, identifier; ORCID, Open Researcher and Contributor; PID, persistent identifier; ROR, Research Organization Registry.

**Table 5:** Open Pharma Vision and roadmap for the FAIRification of company research publication data

Vision for FAIRification of data	
Immediate goal	Agreement across the pharmaceutical industry about applying the FAIR framework for all data associated with human research publications
Ultimate goal	All publication-associated data are FAIR while protecting individual privacy

Stakeholder actions	
Authors of company-sponsored biomedical research	Apply FAIR principles (while protecting patient confidentiality) to any data collected, analysed and/or deposited^30^ Provide a data sharing statement with all (co-)authored articles
Pharmaceutical company funders	Implement FAIR standards for all data associated with publications^30^ Collaborate to achieve cross-industry implementation of FAIR principles while safeguarding patient confidentiality Work together to address current challenges (ethical and pragmatic) in data sharing
Publishers	Standardise data sharing requirements for biomedical research publications Make all requirements aligned with FAIR principles and legal and ethical constraints^30^ Work together to address current challenges in data sharing for all stakeholders

FAIR, Findability, Accessibility, Interoperability, and Reuse.

**Table 6:** Open Pharma Vision and roadmap for involving the patient

Vision for involving the patient	
Immediate goal	100% of human research papers include a statement about if/how the patient perspective was included
Ultimate goal	100% of biomedical research papers include a statement about if/how the patient perspective was integrated in the study and publication concept or plan

Stakeholder actions	
Authors of company-sponsored biomedical research	Make sure that patients and/or their representatives have an opportunity to author research publications when appropriate Contribute to the development of easily understood content types when appropriate
Pharmaceutical company funders	Develop internal guidance on patient inclusion in research, outline appropriate research opportunities and define optimal timing of engagement When relevant and appropriate, give patients and/or their representatives the opportunity to be involved in the research process and/or be authors of company research publications In all published articles, include a statement outlining if/how patients were involved in the study and publication outputs Develop content formats tailored to different audience types and disseminate them through audience-appropriate channels
Publishers	Enable patients and/or their representatives to be authors and reviewers for articles within their expertise (eg, by including a standard affiliation for patients) that would also facilitate bibliometric analyses of patient involvement in publications Tailor journal submission requirements to allow patient authors to specify the nature of their involvement (eg, subject area expert/partner vs participant providing data) Enable ORCID iDs for patient authors and their representatives^28^ Have standardised processes for designating patient contributions Ensure manuscript submission forms are comprehensible to patient authors and/or their representatives Enable multiple content types and disseminate them through audience-appropriate communication channels, including social media

iD, identifier; ORCID iDs, Open Researcher and Contributor iDs; vs, versus.

Ultimately, we believe that publishing peer-reviewed company research publications with open access, including a plain language summary within the published manuscript, making optimal use of metadata, committing to Findability, Accessibility, Interoperability, and Reuse (FAIR) data principles, and proactively engaging patient perspectives in the research and publication process together improve transparency, advance medical science, and have the potential to benefit patient care.

## Open access

Publishing open access ensures that high-quality, peer-reviewed evidence is available to anyone who needs it, anywhere in the world, without paywall restrictions. As funders and conductors of biomedical research,^3 6 7^ pharmaceutical companies hold a leadership responsibility for publishing research that provides transparency, advances medical science, and is most effective in improving patient care.^8^

Here, we reiterate and reinforce the priorities outlined in our 2019 Open Access Position Statement, which made an immediate call for all company-funded research to be free to read from the date of publication.^8^ To avoid narrowing authors’ choice of journal while there remains heterogeneity in the copyright licence types offered across publishers, we support use of any type of open access copyright licence (ie, any Creative Commons (or equivalent) licence variant).^9^

We echo this priority as an immediate step in our vision for company research publications (table 2). To ensure feasibility of this short-term vision, we endorse the use of green open access avenues (ie, self-archiving the author-accepted manuscript in relevant digital repositories (eg, PubMed Central, Europe PMC or institutional sites))^10^ if required to enable immediate and/or wider access than that provided by the journal, but always sharing the Digital Object Identifier (DOI) URL when citing the published article, in line with Crossref recommended practice.^11^ The DOI can be used by open access curators (eg, www.unpaywall.org) to centralise peer-reviewed open access articles irrespective of where they were published or deposited, helping to support research discoverability and transparency.^12^

Looking to the future, however, our ultimate aspiration is that a single version of company-funded research be published online, without paywall restrictions on the journal website from the date of publication and immediately available for reuse (ie, gold open access) (table 2). This ultimate goal embodies the open access definition set out in the foundational Budapest Open Access Initiative Declaration, a definition based on unrestricted, free and immediate access and reuse of peer-reviewed research, achievable by publication under a Creative Commons Attribution (CC BY) licence.^13^ It also extends to company-funded research the same principles—that unrestricted access to biomedical research maximises its potential benefit to health and contributes to better and more efficient science and innovation.^14 15^ These principles underpin open access mandates from funders operating across the biomedical research community, including the Gates Foundation,^16^ the European Commission,^14^ the Wellcome Trust^15^ and the National Institutes of Health.^17^ Some pharmaceutical companies have already mirrored these non-commercial funders by introducing similar open access mandates,^18^,^20^ or commitments to majority open access publication of research.^21^

Open Pharma is committed to working closely with publishers and pharmaceutical companies to achieve this vision and to addressing current inequities in open access publishing models, including global diversity challenges posed by article processing charges, selective licence restrictions imposed by some journals on company-funded research and limited transparency around open access model pricing and permitted terms of content reuse (including what constitutes ‘commercial use’).^22 23^ We believe that publishing practices that prevent company research from being free to read, cite and reuse from the point of publication represent a barrier to equitable access to biomedical research, with negative consequences for patient health.

## Plain language summaries

Researchers, healthcare professionals, policymakers and patients and their caregivers are a few of the stakeholders with agency to influence health outcomes. We believe that anyone who needs to understand peer-reviewed research should be able to do so, regardless of their prior subject knowledge, literacy level or first language. All stakeholders invited to participate in healthcare decision-making should be equipped to do so effectively through stakeholder-appropriate healthcare communications—from time-poor specialists to non-specialist healthcare professionals (eg, general practitioners, pharmacists and nurses) and researchers, journalists, policymakers and the public.

We call for all peer-reviewed company research publications to include a plain language summary: a summary of the research that is understandable, free of technical jargon, unbiased, non-promotional, easily accessed and peer reviewed (as part of the journal’s adjudication of the overall article).^24^ The immediate priority is to implement this goal for all study publications that underpin regulatory decisions on drug (or medical device) approvals; moving forward, it should be extended to all pharma-sponsored biomedical research, agnostic to intervention type (eg, pharmacological, surgical, non-pharmacological) (table 3).

Plain language formats vary from short plain language summaries included as part of the peer-reviewed research publication (PLS) to separate standalone, peer-reviewed articles written in clear, non-technical language (a plain language summary publication (PSLP)). As a minimum, we endorse a text-based PLS format of approximately 250 words within the peer-reviewed publication, as advocated in the Open Pharma consensus recommendations for plain language summaries.^24^ Text-based formats are the most discoverable through indexing in directories such as PubMed, and they are easy to translate. Standardising the format and indexing of plain language summaries can support pathways to wide-scale adoption and the quality and credibility of these accessible summaries. Standardised, text-based plain language summaries can then serve as a basis for development of complementary audience-specific summaries in purpose-appropriate formats, including videos and infographics.

Sharing research through plain language summaries supports research communication across a broad range of audiences and offers the potential to improve the transparency, accountability, accessibility, discoverability, understandability and equity of medical research.^24^ Plain language summaries counteract misinformation and enable a wide and diverse range of people to understand and use high-quality, peer-reviewed healthcare research information.

To maximise the discoverability and benefit of plain language summaries for all audiences, our aspiration is that they appear alongside the manuscript abstract: in the full-text PDF of the publication, in the article HTML on the journal website, and (for indexed journals) on PubMed. Aggregation of plain language summaries within a centralised, dedicated library that includes links to/from the associated full-text manuscript would be a desirable addition. At the time of writing no such repository has been established, but the concept is being explored by relevant publisher stakeholders. In recognition that not all journals currently have processes in place to facilitate immediate inclusion of plain language summaries alongside the abstract, we call for publishers to refine their processes to enable their discoverability in this way and, in the meantime, for authors and funders to request that any plain language summary submitted with their manuscript be peer reviewed and included within the main publication or minimally signposted within its supplementary materials.

## Author and institutional metadata

Transparency in research provenance is critical to research integrity, quality appraisal, impact evaluation and overall trust. In a digital publishing environment, metadata are integral to proof of provenance. Metadata are labels/tags that are assigned to digital files to describe them: what they are (eg, file type and object type), what they contain (eg, title and keywords), when they were generated (eg, date) and who created them (eg, author). Metadata help with file organisation, categorisation, identification and retrieval. For digital objects (articles, images, datasets), metadata can include persistent identifiers (PIDs)—unique and unchanging strings of letters and numbers that serve as a long-lasting reference to a digital object (eg, DOIs for research publications and International Standard Book Numbers for books).

PIDs are an important component of a robust digital content infrastructure; they are easily machine readable and identifiable by online searches and can be integrated across information systems. They make published research easier to find, authenticate and curate. The practice of citing unique study identifiers (eg, National Clinical Trial numbers) within associated research publications has demonstrated the power of PIDs to increase research transparency and cross-referencing.^25^ Originally implemented for trials, the value of prospective registration and citation of PIDs is study-design agnostic; we support the practice for both interventional and non-interventional studies.

Other PIDs relevant to biomedical science publications are those unique to contributing authors (Open Researcher and Contributor (ORCID) iDs), research organisations (Research Organization Registry iDs) and individual grants (grant iDs).^26 27^ Together, PIDs provide structured information (metadata) about the provenance of the research; embedding them in research publications contributes to transparency about the individuals, organisations and funders responsible for the work. ORCID iDs, which any person can have regardless of affiliation (patient authors included)^28^ can eradicate ambiguity about authors with similar names and unify the publication records of authors who have changed names. They can also contribute to increased research impact by linking authors with their wider research portfolio and serving as a digital resume for those interested in future research collaborations.^26^ ORCID iDs also provide a potential route for maintaining up-to-date author disclosures, either within the ORCID platform or via linked disclosure platforms such as Convey.^29^ Beyond the value of PIDs for authentication and discoverability of individual research publications, the use of these metadata creates a digital infrastructure that also enables evaluation of research impact and of research collaborations by, for example, tracing and curating grant PIDs across collaborating organisations, studies and associated publications.

We call for all company research publications to include ORCID iDs for company authors and for improved uptake of research organisation and grant iDs among all authors, contributors and funders (table 4). Our future aspiration is that the metadata of all company research publications will contain iDs for all authors and acknowledged contributors (ORCID iDs), that publishers will support inclusion of research organisation iDs for affiliations of all authors and contributors, and that the metadata of all company and investigator-initiated study publications will contain grant PIDs to optimise triangulation of the information critical to research provenance and trust. We also welcome further innovation from those working in the PID sector to enable increased transparency and traceability of funding disclosures that do not contain a specific grant number and associated PID.

## FAIR data

The 2016 FAIR Principles provide an aspirational framework for the scientific management and stewardship of biomedical research data, focused on the FAIR of digital assets.^30^ Data FAIRification is a challenging but worthwhile endeavour that we support in our commitment to drive positive change in company research publications.

FAIR’s ‘Findability’ and ‘Accessibility’ principles resonate with our own vision for greater use of author and institutional metadata within company research publications, and with our call to extend open access CC BY licence use to all company research publications. FAIR’s ‘Interoperability’ principle underscores the importance of establishing robust and standardised methods of describing, structuring and storing data such that data(sets) can be combined, efficiently transferred across software applications and workflows, and reused, replicated and/or combined in different settings. Achieving interoperability of digital assets ensures that maximum benefit can be derived from their ‘Reuse’ which, in the context of company research publications, is enabled by data sharing.

Data sharing is a core component of Open Science frameworks owing to its potential to enhance research transparency, reproducibility and collaboration, and to reduce research inefficiencies.^231^,^34^ We endorse the European Federation of Pharmaceutical Industries and Associations position that sharing clinical trial information is in the best interests of patients, clinicians and medical research, providing that the information is shared responsibly, taking into account the need to maintain patient confidentiality, maintaining the integrity of regulatory systems worldwide and continuing to support innovation with appropriate arrangements for commercial-in-confidence information.^35^ In this endeavour, we applaud sizeable efforts across the pharmaceutical industry that have increased the transparency of ongoing and completed clinical trials, and enabled access to the participant-level data that underpin trial publications through managed access platforms, such as ClinicalStudyDataRequest.com (CDSR: www.clinicalstudydatarequest.com), Vivli (www.vivli.org) and YODA (https://yoda.yale.edu).^36^ We also support ongoing efforts within biomedical research and publishing to determine how best to operationalise patient-level data sharing for company research publications: those that aim to balance the value derived from responsible data sharing with the resource required to enable them.^36 37^ Publishers also have an important part to play in incentivising ongoing investment in data sharing efforts through their provision of channels for the dissemination of data sharing project outputs. Ultimately, the full promise of data sharing efforts will only be realised through joint support and adherence to data sharing principles by all research funders.

In the pursuit of FAIRification of data supporting company research publications, we call on pharmaceutical companies to build on the efforts of the Pistoia Alliance FAIR implementation toolkit^38^ and to work together to agree on the application of the FAIR framework principles for the human data that underpin their publications (table 5). Once consensus has been achieved, our ultimate aspiration is industry-wide adoption of these principles.

## Patient involvement

The Open Science movement encourages new social actors to engage in the scientific process to help broaden knowledge, address systemic inequalities, fight misinformation and focus on problems of global societal importance.^2^ Improving health outcomes requires research to be discoverable, accessible, understandable and trusted; therefore, it must also be relevant to the needs and lived experiences of patients, carers and patient advocates. Recognition is growing of the value of the patient perspective (or direct patient involvement) in designing and conducting research that is acceptable within current societal norms and meaningful to those living with and/or managing life-changing and life-limiting conditions.^39 40^

Aligned with these views, we believe that meaningful involvement of patients/patient perspectives within human research publications can improve the comprehensibility of the research, increase trust in the findings and add value to their dissemination. For example, including the patient perspective when designing a study can ensure that its endpoints are truly relevant to the population that the research aims to serve, and that pragmatic realities that may increase patient recruitment and retention are taken into consideration. Furthermore, the perspective of patients at the time of research dissemination, whether provided by individuals with lived experience or by their advocates/representatives, helps to frame research findings so that they are appropriately contextualised and interpreted for clinical audiences and/or presented in a manner that is relatable and understood by non-specialist audiences. Where a provision of the patient perspective results in a research contribution that meets the criteria for authorship,^41^ this should be recognised and enabled by research funders, co-authors and publishers, including use of patient-authorship metadata to aid discoverability of patient contributions.^28^ Where the contribution of patient insights is insufficient to constitute authorship, contributions should be appropriately acknowledged (subject to patient permission) in manuscripts and congress presentations.^42 43^

The appropriateness and value of the patient perspective for research and publication planning is study-specific, but we believe that disclosure of the level of involvement (even if none) has intrinsic value. Disclosure contributes to transparency, trust and credibility of research; it provides an opportunity to highlight how patient perspectives may have informed the research and its reporting while simultaneously promoting a research ecosystem more predisposed to patient centricity.

Noting that patient representation and/or involvement is not a quality marker but that disclosure contributes to research transparency, we call for immediate inclusion of a statement in all human study publications outlining the extent to which the patient perspective was included (table 6). In the future, our aspiration is that this practice becomes standard across all medical research publications.

## Conclusion

We appreciate that achieving the Open Pharma Vision will take time and effort, but the results will be worth the endeavour. The aspirations outlined may be challenging to some, but every positive change requires a departure from the status quo, an investment in the future and a commitment to innovation.

Components of the vision will also be challenged, which will present opportunities for discourse and exchange of differing perspectives. Open Science concepts will continue to evolve—optimal communication practices may be refined by new evaluation metrics and the advent of widely accessible generative artificial intelligence, and publishing business models may be subject to change in the pursuit of improved global equity. Critical challenge and iterative refinement are welcomed; they are inherent components of the scientific process, and the vision statements should be similarly revisited and refined. Scientific progress is achieved through positive collaboration and successful partnerships. It is only through collaboration between diverse stakeholders, including commercial organisations, academic institutions, publishers, patients and patient organisations and researchers, that we will be able to co-create solutions, implement course corrections and overcome barriers and legacy inertia. This article outlines the initial iteration of the Open Pharma Vision, but the authors recognise that ongoing collaboration is required to ensure the vision continues to resonate with all stakeholder groups. Reaching a shared vision was a critical first step; identifying barriers to its implementation and potential ways to overcome them must now follow. We call on all stakeholders to realise the Open Pharma Vision, not without appreciation of the efforts required but with aspirations for a more open and trusted future for company research publications that will ultimately deliver greater benefit for patient care and improved global health.
