# Supplementary material for: An Open Pharma Vision for company-sponsored biomedical research publications
Source: BMJ Open. 2026 May 8;16(5):e104128. doi: 10.1136/bmjopen-2025-104128 (PMC13157756; doi:10.1136/bmjopen-2025-104128)
Supplement: Supplementary file 1 [file bmjopen-2025-104128.reviewer_comments.pdf]

PEER REVIEW HISTORY

BMJ Open publishes all reviews undertaken for accepted manuscripts. Reviewers are asked to complete a checklist review form and are provided with free text boxes to elaborate on their assessment. These free text comments are reproduced below.

ARTICLE DETAILS

Title (Provisional)

An Open Pharma vision for company-sponsored biomedical research publications

Authors

Osório, Joana; Baróniková, Slávka; Dews, S A; Mysore, Santosh; Patrikis, Kalliopi; Philippon, Valérie; Rains, Christopher P; Schmidt, Anja; Skobe, Catherine; Winchester, Chris

---

VERSION 1 - REVIEW

**Reviewer**	**1**
**Name**	**Ndosi, Mwidimi**
**Affiliation**	**University of the West of England**
**Date**	**20-Aug-2025**
**COI**	**None**

Thank you for the opportunity to review this communication manuscript presenting a vision for company-sponsored biomedical research publications. I commend the authors for this initiative.

While the concept itself is not entirely novel (as similar principles are already established in publicly funded research), the manuscript is forward-looking for industry-sponsored research and is well written.

The authors acknowledge that the development of this statement was not based on a formal guideline development process. However, it would strengthen the manuscript to provide more detail on how the working group was formed and whether efforts were made to engage major stakeholders in the pharmaceutical industry. This is particularly important as the article calls on all stakeholders to realise the Open Pharma Vision. If such outreach was limited, the authors should describe the group's composition and discuss the potential limitations of the vision due to missing perspectives of other stakeholders.

Although patient involvement is highlighted as a standard in the initiative, patients were not included in developing this vision. The authors can consider whether this omission could undermine the credibility of the vision and whether it is feasible to involve patients at this

stage. If they decide to do this (not ideal but better than never), they can discuss the if any points were modified by patient review 9perspective).

While the vision statements themselves are clear and good, practical implementation remains a key challenge. It may be helpful to include a column in the vision statement tables (for each statemet) specifying potential implementation barriers. Befor the conclusion, please include a discussion section where you could discuss how these barriers could be addressed. This would provide a more actionable roadmap for achieving the Open Pharma Vision.

Also the discussion section could include the key limitations of the process used to develop this vision. This will help readers contextualise the recommendations and understand the scope of the work. Overall, this is a well-written and thoughtful manuscript. Addressing these points would strengthen the paper, enhance its credibility, and make it more actionable for the intended audience.

**Reviewer**	**2**
**Name**	**Crico, Chiara**
**Affiliation**	**Azienda USL-IRCCS di Reggio Emilia, Legal Medicine and Bioethics**
**Date**	**17-Dec-2025**
**COI**	**None**

This is not a research article, rather a call to action for sponsored biomedical research. I think the vision endorsed by the authors has great value in improving research transparency, accessibility and impact on patient care. The goals described in the article are ambitious and challenging but at the same time actionable. Thank you for your contribution.

I have only minor comments:

- line 3 page 13: I would include also studies on non pharmacological interventions, as they may have a deep impact on clinical practice (e.g. studies on radiation therapy or innovative surgical interventions) even though drugs are not involved;

- Table 3, lines 21-22: PLS can be developed easily and in a cost-efficient way with AI sotfwares, not just professional writers

- page 17, "Patient involvement": I believe that it should be specified more clearly how patients should/can be included, because there is a big difference between including expert patients/associations/individual patients as reviewers or to obtain feedback, and including them in the design and development phase of a research programme (the latter being a more consistent method of engaging patients in research).

VERSION 1 - AUTHOR RESPONSE

Reviewer 1

Thank you for the opportunity to review this communication manuscript presenting a vision for company-sponsored biomedical research publications. I commend the authors for this initiative.

While the concept itself is not entirely novel (as similar principles are already established in publicly funded research), the manuscript is forward-looking for industry-sponsored research and is well written.

Overall, this is a well-written and thoughtful manuscript. Addressing these points would strengthen the paper, enhance its credibility, and make it more actionable for the intended audience.

We thank the reviewer for their thoughtful comments about this article; our responses to individual points are outlined in the table below.

Reviewer comment	Author response
The authors acknowledge that the development of this statement was not based on a formal guideline development process. However, it would strengthen the manuscript to provide more detail on how the working group was formed and whether efforts were made to engage major stakeholders in the pharmaceutical industry. This is particularly important as the article calls on all stakeholders to realise the Open Pharma Vision. If such outreach was limited, the authors should describe the group's composition and discuss the potential limitations of the vision due to missing perspectives of other stakeholders.	Thank you for providing this comment and suggesting how we can strengthen the article. To address this, the authors have made changes to the article as outlined in the bullet points below.<br><br>• **Developed figure 1:** this outlines the steps taken towards gaining broad stakeholder consensus on the vision statements prior to the taskforce developing the final vision statements presented within this article.<br>• **Patient and public involvement statement:** included more details of how patients and the public were consulted prior to the article being developed.<br>• **Strengths and limitations section:** we included more detail to the strengths and limitations section of the article (addition of point 1, amends to point 2, addition of points 5 and 6) to outline the stakeholder consensus sought prior to the taskforce developing the vision statements.<br>• **Introduction:** added/amended the following sentences in the

	introduction to further define the roles and experience of the taskforce members.<br>○ *We established a taskforce to define steps to advance transparency in company research publications beyond public disclosure of human trial data, which most pharmaceutical companies have already committed to.*<br>○ *The taskforce comprised senior professionals from the pharmaceutical and medical communications sectors who had extensive experience in scientific and medical publications, medical affairs, patient affairs, and patient advocacy.*<br>○ *Membership of the taskforce was voluntary, and all members were involved in the Open Pharma collaboration.*<br>○ *Following consultation with diverse stakeholders during the 2023 Open Pharma Summit on their aspirations for company research publications (figure 1), the role of the taskforce was to formalise and champion the ambitions they had voiced.*
Although patient involvement is highlighted as a standard in the initiative, patients were not included in developing this vision. The authors can consider whether this omission could undermine the credibility of the vision and whether it is feasible to involve patients at this stage. If they decide to do this (not ideal but better than never), they can discuss the if any points were modified by patient review perspective).	Thank you for providing this comment and highlighting the need to clarify how patients were consulted during the development of the vision statements. To address this, the authors have revised the article as outlined in the bullet points below.<br><br>• **Developed figure 1:** this provides details of the stakeholder groups consulted prior to the vision statements being finalised. Stakeholders included individual patient advocates and representatives from patient organisations.<br>• **Strengths and limitations section:** we included more detail to the

	strengths and limitations section of the article (addition of point 1, amends to point 2, addition of points 5 and 6) to outline the stakeholder consensus sought prior to the taskforce developing the vision statements.<br>• **Conclusions:** we amended the Conclusions section with the following sentence to reiterate that the authors will continue to engage with important stakeholders (including patient representatives) to ensure the vision statements are relevant for all stakeholder groups.<br>○ *This article outlines the initial iteration of the Open Pharma Vision, but the authors recognise that ongoing collaboration is required to ensure the vision continues to resonate with all stakeholder groups. Reaching a shared vision was a critical first step; identifying barriers to its implementation and potential ways to overcome them must now follow.*
While the vision statements themselves are clear and good, practical implementation remains a key challenge. It may be helpful to include a column in the vision statement tables (for each statement) specifying potential implementation barriers. Before the conclusion, please include a discussion section where you could discuss how these barriers could be addressed. This would provide a more actionable roadmap for achieving the Open Pharma Vision.	Thank you for this feedback regarding practical implementation of the vision. The authors carefully considered including implementation strategies and potential barriers during article development. However, it was ultimately decided that the article should remain concise and focus solely on the vision statements. With this in mind, the authors have made amends to the article to address this point as noted in the bullets below.<br><br>• **Strengths and limitations section:** we added point 5 to highlight that implementation barriers have not been discussed in the article.<br>• **Conclusions:** we added the following sentence to note that the authors will review potential implementation barriers that may arise when working towards achieving the vision.

	◦ *Reaching a shared vision was a critical first step; identifying barriers to its implementation and potential ways to overcome them must now follow.*
Also the discussion section could include the key limitations of the process used to develop this vision. This will help readers contextualise the recommendations and understand the scope of the work.	Thank you for this recommendation. As noted above, the authors would like to keep the article concise. However, to address this point, the authors have made the changes outlined in the bullet point below.<br><br>• **Strengths and limitations section:** we included more detail to the Strengths and limitations section (addition of point 1, amends to point 2, addition of points 5 and 6) to outline the stakeholder consensus sought prior to the taskforce developing the vision statements and state that beyond the initial consensus period, patient or the public were not involved in the development of the vision statements.

Reviewer 2

This is not a research article, rather a call to action for sponsored biomedical research. I think the vision endorsed by the authors has great value in improving research transparency, accessibility and impact on patient care. The goals described in the article are ambitious and challenging but at the same time actionable. Thank you for your contribution.

We thank the reviewer for their thoughtful comments about this article; our responses to individual points are outlined in the table below.

Reviewer comment	Author response

Line 3 page 13: I would include also studies on non-pharmacological interventions, as they may have a deep impact on clinical practice (e.g. studies on radiation therapy or innovative surgical interventions) even though drugs are not involved<br><br>*Clarification:*<br>*The comment was referring to line 3 page 14, which is page 13 of 23 of the PDF generated in our submission system. The sentence referred to is as follows:*<br><br>*The immediate priority is to implement this goal for all study publications that underpin regulatory decisions on drug (or medical device) approvals; moving forward, it should be extended to all company biomedical publications (table 3).*	Thank you for this comment; the authors have amended the sentence below to address it.<br><br>• *The immediate priority is to implement this goal for all study publications that underpin regulatory decisions on drug (or medical device) approvals; moving forward, it should be extended to all pharma-sponsored biomedical research, agnostic to intervention type (e.g. pharmacological, surgical, non-pharmacological) (**table 3**).*
Table 3, lines 21–22: PLS can be developed easily and in a cost-efficient way with AI software, not just professional writers	Thank you for this comment; the authors have added a footnote to table 3 to address it.<br><br>• **Additional footnote on Table 3**: *†AI has the potential to provide efficiencies with PLS development; however, it is imperative that the AI-generated PLS be reviewed by a professional medical writer or a representative of the intended non-specialist audience to ensure accuracy and suitability. It is also noteworthy that not all organisations and institutions have access to generative AI capable of PLS development.*

Page 17: "Patient involvement": I believe that it should be specified more clearly how patients should/can be included, because there is a big difference between including expert patients/associations/individual patients as reviewers or to obtain feedback, and including them in the design and development phase of a research programme (the latter being a more consistent method of engaging patients in research).	Thank you for this comment; the authors agree and have made the changes detailed in the bullet points below to address it.<br><br>• **Amends to this sentence:** *Aligned with these views, we believe that meaningful involvement of patients/patient perspectives within human research publications can improve the comprehensibility of the research, increase trust in the findings and add value to their dissemination*<br>• **Added this sentence:** *For example, including the patient perspective when designing a study can ensure that its endpoints are truly relevant to the population that the research aims to serve, and that pragmatic realities that may increase patient recruitment and retention are taken into consideration.*

VERSION 2 - REVIEW

**Reviewer**	**1**
**Name**	**Ndosi, Mwidimi**
**Affiliation**	**University of the West of England**
**Date**	**02-Apr-2026**
**COI**	

The authors have been addressed my previous concerns. While they have not restructured the tables or incorporated a dedicated discussion of implementation, these omissions are now acknowledged within the revised Strengths and Limitations section. I believe this is transparent enough for readers to contextualise the vision.

I also welcome the addition of Figure 1 and the clarification of the patient and public involvement statement, both of which improve the overall clarity of this communication manuscript.
